# C‐PLAN index as a prognostic factor for patients with previously untreated advanced non‐small cell lung cancer who received combination immunotherapy: A multicenter retrospective study

**DOI:** 10.1111/1759-7714.14798

**Published:** 2023-01-12

**Authors:** Kei Sonehara, Ryota Ozawa, Mineyuki Hama, Shuhei Nozawa, Toshihiko Agatsuma, Kenichi Nishie, Akane Kato, Akemi Matsuo, Taisuke Araki, Masamichi Komatsu, Kazunari Tateishi, Masayuki Hanaoka

**Affiliations:** ^1^ First Department of Internal Medicine Shinshu University School of Medicine Matsumoto Japan; ^2^ Department of Respiratory Medicine Nagano Red Cross Hospital Nagano Japan; ^3^ Department of Respiratory Medicine Suwa Red Cross Hospital Suwa Japan; ^4^ Department of Respiratory Medicine Nagano Municipal Hospital Nagano Japan; ^5^ Department of Respiratory Medicine National Hospital Organization Shinshu Ueda Medical Center Ueda Japan; ^6^ Department of Respiratory Medicine Iida Municipal Hospital Iida Japan; ^7^ Department of Respiratory Medicine Ina Central Hospital Ina Japan; ^8^ Department of Respiratory Medicine, Minaminagano Medical Center Shinonoi General Hospital Nagano Japan

**Keywords:** combination immunotherapy, C‐PLAN index, non‐small cell lung cancer, prognostic factor

## Abstract

**Background:**

Combination immunotherapy (immune checkpoint inhibitors and cytotoxic anticancer agents) is widely used as first‐line treatment for advanced non‐small cell lung cancer (NSCLC). However, the therapeutic effect of combination immunotherapy has not been fully investigated. C‐reactive protein, performance status, lactate dehydrogenase, albumin, and derived neutrophil‐to‐lymphocyte ratio (C‐PLAN) are useful biomarkers for predicting the prognosis of NSCLC; however, there are no reports examining the C‐PLAN index, which combines these five factors in a single prognostic factor.

**Methods:**

We retrospectively collected data from 178 patients with previously untreated advanced NSCLC who received combination immunotherapy at multicenter institutions in Nagano Prefecture between December 2018 and April 2022. We investigated the utility of the C‐PLAN index as a prognostic factor using Cox regression analysis and correlated it with survival.

**Results:**

The good and poor C‐PLAN index groups included 85 and 93 patients, respectively. The good C‐PLAN index group had a longer median progression‐free survival (PFS) (10.7 vs. 6.0 months; *p* = 0.022) and overall survival (OS) (25.3 vs. 16.5 months; *p* = 0.003) than the poor C‐PLAN index group. The C‐PLAN index was an independent favorable prognostic factor that correlated with PFS and OS in multivariate analysis. The good C‐PLAN index group had a higher proportion of never‐smokers (16.5 vs. 4.3%; *p* = 0.007) and stage III disease/postoperative recurrence (32.9 vs. 15.1%; *p* = 0.005) than the poor C‐PLAN index group.

**Conclusion:**

The C‐PLAN index is a useful prognostic factor for patients with previously untreated advanced NSCLC undergoing combination immunotherapy.

## Graphical abstract

The C‐PLAN index, which combines CRP, PS, LDH, Alb, and derived NLR, may be a useful biomarker that reflects the prognosis of patients with advanced NSCLC who received combination immunotherapy. The OS for the good C‐PLAN index group were significantly longer than those for the poor C‐PLAN index group (25.3 [16.7–33.9] vs. 16.5 [12.2–20.8] months, *p* = 0.003).

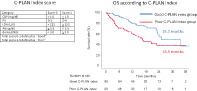

## INTRODUCTION

The first‐line treatment for advanced non‐small cell lung cancer (NSCLC) has changed with the approval of immune checkpoint inhibitors (ICIs), such as anti‐programmed death 1 (PD‐1) antibody and anti‐programmed death ligand 1 (PD‐L1) antibody. Recently, in addition to ICI monotherapy, combination immunotherapy (ICIs and cytotoxic anticancer agents; anti‐PD‐1 and anticytotoxic‐T‐lymphocyte‐associated protein 4 (CTLA‐4) antibodies; and anti‐PD‐1 antibody, anti‐CTLA‐4 antibody, and cytotoxic anticancer agents) is recommended for previously untreated NSCLC, which has further improved the prognosis of advanced NSCLC.^1^, ^2^, ^3^, ^4^, ^5^, ^6^, ^7^ Several studies have investigated biomarkers that predict the efficacy of chemotherapy or immunotherapy for patients with advanced NSCLC.^8^, ^9^

PD‐L1 expression in tumor cells is the most common biomarker.^1^, ^2^, ^3^, ^4^, ^5^, ^6^, ^7^ However, ICIs may be ineffective, even in patients with NSCLC with high PD‐L1 expression in tumor cells.^10^ Therefore, in clinical practice, there is a need to identify simple and reliable biomarkers.

Studies have reported that potential biomarkers reflecting systemic nutrition and inflammation status, such as neutrophil‐to‐lymphocyte ratio (NLR) and platelet‐to‐lymphocyte ratio, are useful biomarkers that reflect the prognosis of patients with NSCLC who received nivolumab.^9^ The modified Glasgow Prognostic Score (mGPS) and lung immune prognostic index (LIPI) were also evaluated as predictors for the prognosis of lung cancer.^11^, ^12^, ^13^ The mGPS is a combination of C‐reactive protein (CRP) ≥ 1.0 mg/dl and serum albumin (Alb) ≥ 3.5 g/dl. The LIPI is a combination of a derived NLR (dNLR) > 3 and serum lactate dehydrogenase (LDH) levels higher than the upper normal limit. The mGPS and LIPI can be classified into three groups according to each score. These biomarkers are easily measurable, have clear cutoff values, and are useful prognostic factors for lung cancer.^11^, ^12^, ^13^ However, these markers do not include performance status (PS), which reflects a patient's general condition. A previous study reported that PS was the most significant prognostic factor that correlated with survival in patients with NSCLC who received ICIs.^14^

To our knowledge, there have been no reports combining the mGPS, LIPI, and PS to evaluate the prognosis of patients with NSCLC who received combination immunotherapy. The C‐PLAN index, which combines CRP, PS, LDH, Alb, and dNLR, may be a useful biomarker that reflects the prognosis of patients with advanced NSCLC who received combination immunotherapy. In this multicenter retrospective study, we investigated the value of the C‐PLAN index as a prognostic factor for patients with NSCLC who received combination immunotherapy.

## METHODS

### Study design

This multicenter retrospective study included data from 178 patients with previously untreated advanced NSCLC. Patients with NSCLC with sensitizing epidermal growth factor receptor mutations or anaplastic lymphoma kinase translocations were excluded. All patients received combination immunotherapy at multicenter institutions in Nagano Prefecture between December 2018 and April 2022. The study protocol was approved by the Institutional Ethics Review Committee of Shinshu University School of Medicine (approval number: 5494). All relevant data were extracted from electronic medical records in accordance with the principles of the Declaration of Helsinki. An opt‐out method was used wherein patients could decline to participate by filling out a form that was available on our institution's official website. Thus, the need for written informed consent was waived. All data were anonymized.

### Data collection and analysis

Clinical findings were evaluated on the induction of first‐line combination immunotherapy. The tumor response to combination immunotherapy was assessed using the Response Evaluation Criteria in Solid Tumors (version 1.1). The C‐PLAN index scores are shown in Table 1. The cutoff values for CRP, LDH, Alb, and dNLR were set at 1.0 mg/dl, 223 U/l, 3.5 g/dl, and 3, respectively. The cutoff value for PS was 1. The C‐PLAN index score ranged from 0 to 5, with a median of 2. A C‐PLAN index score of 0–1 was defined as good and 2–5 as poor.

**TABLE 1:** C‐PLAN index score

Category	Score 0	Score 1
CRP (mg/dl)	<1.0	≥1.0
PS	0–1	2–4
LDH (U/l)	<223	≥223
Alb (g/dl)	≥3.5	<3.5
derived NLR	<3.0	≥3.0
Total score 0–1 defined as “Good”		
Total score 2–5 defined as “Poor”		

Abbreviations: C‐PLAN, C‐reactive protein, performance status, lactate dehydrogenase, albumin, and derived neutrophil‐to‐lymphocyte ratio, CRP, C‐reactive protein; PS, performance status; Alb, albumin; LDH, lactate dehydrogenase; NLR, neutrophil‐to‐lymphocyte ratio.

The objective response rate (ORR) was defined as complete or partial response. The disease control rate (DCR) was defined as the ORR and stable disease. Overall survival (OS) and progression‐free survival (PFS) were defined from the induction of first‐line combination immunotherapy to the date of death from any cause or last follow‐up and the date of progressive disease (PD), respectively. Clinical findings and survival were compared between the good and poor C‐PLAN index groups.

### Statistical analysis

The cutoff date was June 30, 2022. Mann–Whitney U test and Fisher's exact test were used to compare continuous and categorical variables between the good and poor C‐PLAN index groups. Survival curves were analyzed using the Kaplan–Meier method and compared using the log‐rank test. Cox regression analysis was used to identify independent prognostic factors that correlated with survival. Univariate analysis was performed according to age (<75 vs. ≥75 years), sex (male vs. female), PS (0–1 vs. 2–3), body mass index (<22 vs. ≥22 kg/m^2^), smoking history (never vs. current/former), histological subtype (nonsquamous vs. squamous), PD‐L1 tumor proportion score (TPS) (≥50 vs. <50%), stage (III/recurrence vs. IV), and the C‐PLAN index (good vs. poor). Significant variables (*p* < 0.05) in the univariate analysis were included in the multivariate analysis. Multivariate analysis excluded the effects of confounding variables. SPSS Statistics (version 26; IBM Corp.) was used for statistical analysis.

## RESULTS

### Patient characteristics

The patient characteristics are shown in Table 2. There were 136 patients (76.4%) aged <75 years and 42 patients (23.6%) aged ≥75 years. A total of 148 (83.1%) and 30 (16.9%) patients were male and female, respectively. The histological subtype was nonsquamous in 127 patients (71.3%) and squamous in 51 patients (28.7%). According to the Tumor–Node–Metastasis Classification of Malignant Tumors (eighth edition), 11 patients (6.2%) had stage III disease; 62 (34.8%), stage IVA disease; 74 (41.6%), stage IVB disease; and 31 (17.4%), postoperative recurrence.

**TABLE 2:** Patient characteristics

Category	All patients, *N* (%)
Patients, (*N*)	178
Age, years	
<75/≥75	136 (76.4)/42 (23.6)
Gender	
male/female	148 (83.1)/30 (16.9)
ECOG performance status	
0–1/2–3	154 (86.5)/24 (13.5)
Body mass index	
<22/≥22	93 (52.2)/85 (47.8)
Smoking history	
current and former/never	160 (89.9)/18 (10.1)
Histological subtype	
nonsquamous/squamous	127 (71.3)/51 (28.7)
PD‐L1 TPS	
≥50/1–49/0/unknown	45 (25.3)/61 (34.3) 47 (26.4)/25 (14.0)
Staging	
III/IVA/IVB/postoperative recurrence	11 (6.2)/62 (34.8)/74 (41.6)/31 (17.4)
Laboratory findings, median (range)	
Alb (g/dl)	3.6 (1.6–4.7)
CRP (mg/dl)	1.3 (0.01–31.97)
LDH (U/l)	197 (127–4194)
derived NLR	2.57 (0.70–9.75)
Type of combination therapy	
Platinum + pemetrexed + pembrolizumab	80 (44.9)
Carboplatin + pemetrexed + atezolizumab	4 (2.2)
Carboplatin + nab‐paclitaxel + atezolizumab	3 (1.7)
Carboplatin + paclitaxel + bevacizumab + atezolizumab	11 (6.2)
Platinum + nab‐paclitaxel (paclitaxel) + pembrolizumab	55 (30.9)
Carboplatin + pemetrexed + nivolumab + ipilimumab	12 (6.7)
Carboplatin + paclitaxel + nivolumab + ipilimumab	5 (2.8)
Nivolumab + ipilimumab	8 (4.5)

Abbreviations: Alb, albumin; CRP, C‐reactive protein; ECOG, Eastern Cooperative Oncology Group; LDH, lactate dehydrogenase; NLR, neutrophil‐to‐lymphocyte ratio; PD‐L1, programmed death ligand 1; TPS, tumor proportion score.

### Treatment regimen

Combination immunotherapy involved platinum‐pemetrexed + pembrolizumab for 80 patients (44.9%), carboplatin + pemetrexed + atezolizumab for four patients (2.2%), carboplatin + nab‐paclitaxel + atezolizumab for three patients (1.7%), carboplatin + paclitaxel + bevacizumab + atezolizumab for 11 patients (6.2%), platinum + nab‐paclitaxel/paclitaxel + pembrolizumab for 55 patients (30.9%), carboplatin + pemetrexed + nivolumab + ipilimumab for 12 patients (6.7%), carboplatin + paclitaxel + nivolumab + ipilimumab for five patients (2.8%), and nivolumab + ipilimumab for eight patients (4.5%).

### Efficacy

**FIGURE 1:**
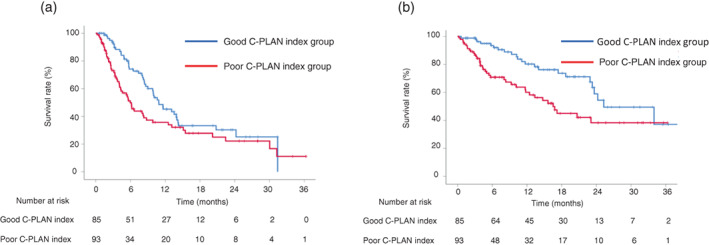
Kaplan–Meier survival curves for patients with non‐small cell lung cancer (NSCLC) with a good and poor C‐PLAN index. (a) The median progression‐free survival (PFS) in the good C‐PLAN index group was significantly longer than that in the poor C‐PLAN index group (10.9 vs. 6.0 months, *p* = 0.022). (b) The median overall survival (OS) in the good C‐PLAN index group was significantly longer than that in the poor C‐PLAN index group (25.3 vs. 16.5 months, *p* = 0.003). C‐PLAN, C‐reactive protein, performance status, lactate dehydrogenase, albumin, and derived neutrophil‐to‐lymphocyte ratio.

The efficacy of first‐line combination immunotherapy in the good and poor C‐PLAN index groups is shown in Table 3. The good and poor C‐PLAN index groups included 85 and 93 patients, respectively. The ORR (95% confidence interval [CI]) for all patients, patients in the good C‐PLAN index group, and patients in the poor C‐PLAN index group were 60.5% (53.0%–67.9%), 62.0% (51.3%–72.8%), and 59.1% (48.8%–69.4%), respectively. The DCR (95% CI) for all patients, patients in the good C‐PLAN index group, and patients in the poor C‐PLAN index group were 86.2% (81.0%–91.5%), 92.4% (86.5%–98.3%), and 80.7% (72.4%–89.0%), respectively. Regarding the ORR, there was no significant difference between the good and poor C‐PLAN index groups (*p* = 0.699). Regarding the DCR, the good C‐PLAN index group had a significantly higher DCR than the poor C‐PLAN index group (*p* = 0.028). The median PFS (95% CI) for all patients, patients in the good C‐PLAN index group, and patients in the poor C‐PLAN index group were 8.8 (7.3–10.3), 10.9 (7.7–14.1), and 6.0 (4.7–7.2) months, respectively. The median OS (95% CI) for all patients, patients in the good C‐PLAN index group, and patients in the poor C‐PLAN index group were 23.6 (19.6–27.7), 25.3 (16.7–33.9), and 16.5 (12.2–20.8) months, respectively. The PFS (*p* = 0.022) and OS (*p* = 0.003) for the good C‐PLAN index group were significantly longer than those for the poor C‐PLAN index group (Figure 1). The 1‐ and 2‐year PFS for the good C‐PLAN index group were 45.2% and 30.3%, respectively. In contrast, the 1‐ and 2‐year PFS for the poor C‐PLAN index group were 35.7% and 22.3%, respectively. The 1‐ and 2‐year OS for the good C‐PLAN index group were 80.2% and 58.9%, respectively. In contrast, the 1‐ and 2‐year OS for the poor C‐PLAN index group were 60.1% and 38.3%, respectively.

**TABLE 3:** Efficacy of first‐line combination immunotherapy in the good and poor C‐PLAN index groups

Efficacy	All patients, *N* (%)	Good C‐PLAN index group, *N* (%)	Poor C‐PLAN index group, *N* (%)	*p*‐value
CR	6 (3.4)	6 (7.1)	0 (0.0)	
PR	95 (53.4)	43 (50.6)	52 (55.9)	
SD	43 (24.2)	24 (28.2)	19 (20.4)	
PD	23 (12.9)	6 (7.1)	17 (18.3)	
NE	11 (6.2)	6 (7.1)	5 (5.4)	
ORR, % (95%. CI)	60.5 (53.0–67.9)	62.0 (51.3–72.8)	59.1 (48.8–69.4)	0.699
DCR, % (95%, CI)	86.2 (81.0–91.5)	92.4 (86.5–98.3)	80.7 (72.4–89.0)	0.028

Abbreviations: CI, confidence interval; C‐PLAN, C‐reactive protein, performance status, lactate dehydrogenase, albumin, and derived neutrophil‐to‐lymphocyte ratio; CR, complete response; DCR, disease control rate; NE, not evaluable; ORR, overall response rate; PD, progressive disease; PR, partial response; SD, stable disease.

### Prognostic factors correlated with survival

Multivariate analysis showed that age < 75 years (hazard ratio [HR], 1.75 [95% CI: 1.20–4.41]; *p* = 0.035), PD‐L1 TPS ≥50% (HR, 1.85 [95% CI: 1.14–3.01]; *p* = 0.013), and a good C‐PLAN index (HR, 1.66 [95% CI: 1.07–2.58]; *p* = 0.025) were independent favorable prognostic factors for PFS (Table 4). Multivariate analysis also showed that stage III disease/postoperative recurrence (HR, 2.82 [95% CI: 1.20–6.62]; *p* = 0.017) and a good C‐PLAN index (HR, 1.87 [95% CI: 1.11–3.15]; *p* = 0.019) were independent favorable prognostic factors for OS (Table 5).

**TABLE 4:** Univariate and multivariate analyses of prognostic factors for progression‐free survival

Category	PFS (months)	Univariate			Multivariate		
		HR	95% CI	*p*‐value	HR	95% CI	*p*‐value
Age, years							
<75 vs. ≥75	10.1 vs. 5.6	1.67	1.07–2.59	0.024	1.75	1.20–4.41	0.035
Gender							
male vs. female	9.9 vs. 7.4	1.08	0.65–1.78	0.775			
ECOG performance status							
0–1 vs. 2–3	9.0 vs. 5.6	1.12	0.64–1.98	0.687			
Body mass index							
<22 vs. ≥22	9.8 vs. 8.6	0.93	0.63–1.37	0.704			
Smoking history							
never vs. current and former	8.4 vs. 8.8	0.84	0.47–1.51	0.560			
Histological subtype							
nonsquamous vs. squamous	8.8 vs. 8.6	1.04	0.67–1.59	0.877			
PD‐L1 TPS (*N* = 153)							
≥50 vs. <50	15.5 vs. 8.3	1.60	1.00–2.58	0.051	1.85	1.14–3.01	0.013
Staging							
III and postoperative recurrence vs. IV	13.1 vs. 8.2	1.63	0.99–2.68	0.056	1.52	0.90–2.57	0.116
C‐PLAN index							
Good (0–1) vs. Poor (2–5)	10.9 vs. 6.0	1.58	1.07–2.33	0.022	1.66	1.07–2.58	0.025

Abbreviations: CI, confidence interval; C‐PLAN, C‐reactive protein, performance status, lactate dehydrogenase, albumin, and derived neutrophil‐to‐lymphocyte ratio; ECOG, Eastern Cooperative Oncology Group; HR, hazard ratio; PFS, progression‐free survival; TPS, tumor proportion score.

**TABLE 5:** Univariate and multivariate analyses of prognostic factors for overall survival

Category	OS (months)	Univariate			Multivariate		
		HR	95% CI	*p*‐value	HR	95% CI	*p*‐value
Age, years							
<75 vs. ≥75	23.3 vs. 25.3	1.14	0.63–2.08	0.670			
Gender							
male vs. female	25.3 vs. 23.6	1.04	0.55–1.96	0.905			
ECOG performance status							
0–1 vs. 2–3	23.6 vs. 16.5	1.53	0.78–3.03	0.213			
Body mass index							
<22 vs. ≥22	23.3 vs. 24.2	0.86	0.52–1.42	0.563			
Smoking history							
never vs. current and former	22.9 vs. 24.2	0.85	0.41–1.80	0.676			
Histological subtype							
nonsquamous vs. squamous	23.3 vs. 34.0	0.75	0.41–1.36	0.337			
PD‐L1 TPS (*N* = 153)							
≥50 vs. <50	NR vs. 23.3	1.40	0.77–2.54	0.264			
Staging							
III and postoperative recurrence vs. IV	NR vs. 20.7	3.27	1.41–7.60	0.006	2.82	1.20–6.62	0.017
C‐PLAN index							
Good (0–1) vs. Poor (2, 3, 4, 5)	26.3 vs. 16.5	2.14	1.28–3.59	0.004	1.87	1.11–3.15	0.019

Abbreviations: CI, confidence interval; C‐PLAN, C‐reactive protein, performance status, lactate dehydrogenase, albumin, and derived neutrophil‐to‐lymphocyte ratio; ECOG, Eastern Cooperative Oncology Group; HR, hazard ratio; OS, overall survival; PD‐L1, programmed death ligand 1; TPS, tumor proportion score.

### Patient characteristics by C‐PLAN index

The good C‐PLAN index group had a higher proportion of never‐smokers (16.5 vs. 4.3%, *p* = 0.007) and stage III disease/postoperative recurrence (32.9 vs. 15.1%, *p* = 0.005) than the poor C‐PLAN index group (Table 6).

**TABLE 6:** Patient characteristics in the good and poor C‐PLAN index groups

Category	Good C‐PLAN index group, *N* (%)	Poor C‐PLAN index group, *N* (%)	*p*‐value
Patients	85	93	
Age, years			
<75/≥75	62 (72.9)/23 (27.1)	74 (79.6)/19 (20.4)	0.298
Gender			
male/female	67 (78.8)/18 (21.2)	81 (87.1)/12 (12.9)	0.076
Body mass index			
<22/≥22	41 (48.2)/44 (51.8)	52 (55.9)/41 (44.1)	0.306
Smoking history			
never/current and former	14 (16.5)/71 (83.5)	4 (4.3)/89 (95.7)	0.007
Histological subtype			
nonsquamous/squamous	64 (75.3)/21 (24.7)	63 (67.7)/30 (32.3)	0.266
PD‐L1 TPS (*N* = 153)			
≥50/<50	17 (23.6)/55 (76.4)	30 (37.0)/51 (63.0)	0.072
Staging			
III and postoperative recurrence/IV	28 (32.9)/57 (67.1)	14 (15.1)/79 (84.9)	0.005

Abbreviations: C‐PLAN, C‐reactive protein, performance status, lactate dehydrogenase, albumin, and derived neutrophil‐to‐lymphocyte ratio; ECOG, Eastern Cooperative Oncology Group; PD‐L1, programmed death ligand 1; TPS, tumor proportion score.

## DISCUSSION

This study showed that the C‐PLAN index is a useful prognostic factor for patients with NSCLC who received combination immunotherapy. Compared to a previous clinical trial,^15^ the present study population was older, with a median age of 70 years (range, 45–83 years); however, survival was comparable to the KEYNOTE‐189 trial (PFS, 9.0 months; OS, 22.0 months). Thus, the real‐world data from our multicenter retrospective study are reliable in clinical practice. Host–tumor interactions affect systemic nutrition and inflammation status. Cancer cachexia, a condition associated with cancer‐induced weight loss, increases the incidence of toxicity and decreases the efficacy of multidisciplinary cancer treatment.^16^ Morimoto et al.^17^ reported that the PFS of patients with NSCLC who received chemoimmunotherapy without cancer cachexia was significantly longer than that of those with cancer cachexia (9.3 vs. 6.7 months) (HR, 1.49 [95% CI: 1.01–2.19]; *p* = 0.004). Thus, a biomarker that accurately assesses the systemic nutrition and inflammation status of the host could improve adherence to combination immunotherapy and appropriate patient selection.

The proposed C‐PLAN index combines five factors: CRP, PS, LDH, Alb, and dNLR. The cutoff values of blood biochemical parameters are the same as those of existing biomarkers, such as the mGPS and LIPI. The components of the mGPS and LIPI are useful biomarkers for predicting therapeutic effects in NSCLC. A GPS of 0–2 (0, good; 1, intermediate; 2, poor), a biomarker first proposed in 2003 for NSCLC, was reported to be a better prognostic marker than PS and stage.^18^ There are few reports on the value of the GPS for patients with NSCLC who received immunotherapy. Kasahara et al.^19^ reported that the post‐treatment GPS (0–1) was significantly correlated with PFS in patients with NSCLC who received anti‐PD‐1 antibody. The LIPI was first proposed in 2018, and has attracted attention as a prognostic factor for patients with NSCLC who received immunotherapy.^12^ PFS and OS differed significantly across LIPI categories (good, intermediate, and poor), following subgroup analysis of the IMPower‐150 trial,^20^ in patients with nonsquamous NSCLC who received carboplatin + paclitaxel + bevacizumab + atezolizumab. The PFS for the good, intermediate, and poor LIPI groups was 12, 8, and 4 months, respectively (*p* < 0.001). In contrast, the OS for the good, intermediate, and poor LIPI groups was 24, 16, and 7 months, respectively (*p* < 0.001). Tumor necrosis factor‐alpha and interleukin‐6 are inflammatory cytokines that promote cancer cell proliferation and progression; however, each cytokine has a different mechanism of action.^21^ Thus, a single index is limited in overcoming patient heterogeneity. The C‐PLAN index can be easily measured using the mGPS (Alb and CRP), LIPI (LDH and dNLR), and PS, and can be compared with a single biomarker to accurately evaluate systemic inflammation and nutrition.

In this study, the good C‐PLAN index group had a significantly longer PFS and OS than the poor C‐PLAN index group. The C‐PLAN index was an independent prognostic factor that correlated with PFS and OS. Regarding PFS, age (<75 years) and PD‐L1 TPS (≥50%), in addition to a good C‐PLAN index, were independent favorable prognostic factors. These results were comparable to those of a previous clinical trial.^1^ The good C‐PLAN index group had a significantly higher DCR than the poor C‐PLAN index group, suggesting that a lower PD rate was a factor for prolonged PFS. The good C‐PLAN index group had a significantly lower PD rate than the poor C‐PLAN index group (7.6% vs. 19.3%; *p* = 0.023). First‐line pembrolizumab or atezolizumab monotherapy is an effective regimen in patients with NSCLC with high PD‐L1 expression in tumor cells; however, it is unclear whether ICI monotherapy or combination immunotherapy is beneficial.^10^, ^22^ Among patients with NSCLC with high PD‐L1 expression, combination immunotherapy should be selected for those with a good C‐PLAN index, considering that approximately 30% of patients with NSCLC who receive ICI monotherapy develop PD within 3 months. The C‐PLAN index was an independent prognostic factor for PFS and OS in the present study; thus, it would be a useful index in clinical practice. Compared to the poor C‐PLAN index group, the good C‐PLAN index group had a significantly higher proportion of never‐smokers and stage III disease/postoperative recurrence. While the high rate of stage III disease/postoperative recurrence is obvious, the high rate of never‐smokers is difficult to interpret. Popat et al.^23^ reported that ever‐smokers who received first‐line pembrolizumab monotherapy had a significantly longer OS (12.8 vs. 6.5 months) (HR, 1.69 [95% CI: 0.5–0.95]) than never‐smokers. Moreover, the OS of ever‐smokers who received platinum‐based chemotherapy was significantly shorter than that of never‐smokers (HR, 1.2 [95% CI: 1.07–1.33]).^23^ It is necessary to investigate the association between smoking history and the efficacy of chemoimmunotherapy in patients with NSCLC in a larger cohort.

This study has several limitations. The sample size is small, and it has a retrospective design. Further, there could be selection bias because the choice of regimen was at the attending physician's discretion and was not fixed beforehand.

In conclusion, the present study indicated that the C‐PLAN index is a useful prognostic factor that correlated with survival in patients with NSCLC who received combination immunotherapy. These results are useful for accurate prognostic assessment and appropriate regimen selection.

## AUTHOR CONTRIBUTIONS

Kei Sonehara wrote and reviewed the manuscript. All authors collected and analyzed the data.

## FUNDING INFORMATION

This research did not receive any specific grant from funding agencies in the public, commercial, or not‐for‐profit sectors.

## CONFLICT OF INTEREST

The authors declare no conflict of interest.
